# Factors Affecting the Incidence and Severity of Oral Mucositis Following Hematopoietic Stem Cell Transplantation

**Published:** 2018-04-01

**Authors:** Maryam Valeh, Mona Kargar, Ava Mansouri, Hosein Kamranzadeh, Kheirollah Gholami, Kazem Heidari, Moluk Hajibabaei

**Affiliations:** 1 School of Pharmacy, Tehran University of Medical Sciences, Tehran, Iran; 2 Research Center for Rational Use of Drugs, Tehran University of Medical Sciences, Tehran, Iran; 3 Hematology-Oncology and Stem Cell Research Center, Tehran University of Medical Sciences, Tehran, Iran

**Keywords:** Oral Mucositis (OM), Nystatin, Povidone iodine, Amphotericin, Chlorhexidin

## Abstract

**Background:** Patients who receive hematopoietic stem cell transplantation (HSCT) experience several complications that oral mucositis (OM) is a frequent symptom. This study was designed to evaluate the incidence, risk factors, prophylaxis and treatment strategies for established OM.

**Materials and Methods: **We included 173 adult patients who received autologous or allogeneic hematopoietic stem cell transplantation in this study. The World Health Organization oral toxicity scale was used to assess the severity of OM. Patients received two prophylactic regimens: regimen 1 contained nystatin, chlorhexidine, povidone iodine and amphotericin B. Regimen 2 contained nystatin and povidone iodine. 70 patients (40.5%) received the first prophylaxis regimen, 89 patients (51.4%) received the second prophylaxis regimen and the remaining 14 patients (8.1%) were not adherence to the use of the mouthwashes and were excluded from the analysis.

**Results:** OM was detected in 60.7% of patients with mean (SD) age of 38.1±14.6 years. Multivariate analysis showed that only the female gender and the prophylactic regimen were the significant predictors of OM.

**Conclusion:** We found that addition of amphotericin B and chlorhexidine, to the nystatin and povidone iodine resulted in a significant beneficial effect in prevention OM.

## Introduction

 Today for many patients suffering from hematopoietic disorders or malignancies that are sensitive to either chemotherapy, radiotherapy or immunotherapy, hematopoietic stem cell transplantation (HSCT) is a standard option^   1 ^. For this treatment, the hematopoietic stem cells that are multipotent and can be obtained from different sources have the main role^   2 ^. However, patients experience a wide variety of toxicities and adverse effects due to the high dose chemotherapy conditioning regimens which should be administered before the HSCT^   2 ^. Among these side effects, gastrointestinal (GI) disorders frequently occur and are associated with nonspecific symptoms^   3 ^. Compared to other adverse effects of treatment with anti-cancer agents, oral mucositis (OM) is the most severe complication based on the patients’ reports^   4 ^. This was also reported in a survey on patients receiving HSCT in which mucositis was accounted for the most considerable adverse effect of HSCT in 42% of patients^   5 ^. This is expected since the high proliferation rates of mucosal cells make them one of the targets of anti-cancer agents^   6 ^. OM can be the consequence of different treatment modalities such as HSCT, radiotherapy, chemotherapy, and molecularly targeted therapy^   4 ^. Mucositis is generally defined as an inflammation in mucosal cells^   7 ^, presenting with erythema or ulcers  ^7^^, ^^8^  due to chemo/radiotherapy ^   7 ^. 

Mucositis is a frequent symptom^8^; however, its frequency depends on different factors. It has been proposed that the diagnosis of patients as well as the age and oral health play a role^   9 ^. Additionally, the characteristics of drugs (type, dose and frequency of administration) are among the influential factors^   9 ^. For example, the incidence of mucositis and its severity is higher in patients taking antimetabolites and alkylating agents ^   5 ^ and busulfan, etoposide, melphalan and methotrexate are commonly associated with OM^   10 ^. Moreover, in patients receiving HSCT, the genetic factor, higher body mass index and receiving total body irradiation as a part of conditioning regimen are also among the risk factors^   5 ^. Moreover, factors such as infections and trauma can aggravate the symptoms^   7 ^. The incidence of OM among patients receiving HSCT varies extensively and can be seen in 75 to 99% of cases in both autologous and allogeneic HSCT^   8 ^. OM is also an important issue in the treatment process. It should be noted that the mucosal damage is a dose-limiting adverse effect for patients receiving conditioning regimen for HSCT^   6 ^. OM is still one of the main problems following high-dose chemotherapy although different prophylactic agents are administered before chemotherapy initiation^   11 ^. 

Mucositis can lead to considerable complications, among which increased gingival and mucosal bleeding and more infections (gingivitis and candidiasis) can be named^   5 ^. Moreover, the disruption in mucosa can lead to oral inconvenience^   12 ^, pain ^4^^,^^8^^,^^12^ , difficulty eating ^4^^,^^12^ , and even water and electrolytes imbalance^   4 ^, as well as increased treatment cost, severe infections and prolonged hospitalization^   12 ^. This disorder can also affect the quality of life^   4 ^ and even speaking^   8 ^ negatively. Severe forms of OM require special medical attention such as providing parenteral nutrition ^13^^,^^14^ , infection prophylaxis^   13 ^ and fluid replacement  ^13^^, ^^14^ . 

To prevent OM, several different mouthwashes and mouth rinses have been tried for patients. Despite the considerable impact of this complication as mentioned above, we currently do not have any prophylactic measures that can completely prevent OM^   15 ^. Only some of the prophylactic agents such as cryotherapy (ice chips) and keratinocyte growth factor (palifermin®) were found to have beneficial effects^   12 ^. Additionally, in terms of treatment, the suggested options for this complication are primarily palliative ^16^^-^^18^ . Different pain relieving agents based on the severity of the symptoms have been suggested to diminish the patients’ pain and discomfort due to OM^   19 ^. Saline mouthwash, ice chips and mouthwashes with anaesthetics are among the agents used for the management of OM^   13 ^. Due to the importance of OM, and -to the best of our knowledge- lack of data regarding the incidence of OM, prophylactic and management strategies in Iran, we designed the present study. The aim of this study was to investigate the current prophylaxis protocols, incidence of OM and its severity along with effective factors and the treatment strategies for established OM in the main HSCT centre in Iran. 

## MATERIALS AND METHODS

 This prospective cohort study was conducted from July 2015 to June 2016 on patients who received HSCT at the Haematology-Oncology and Stem Cell Transplantation Research Centre, Shariati Hospital, Tehran University of Medical Sciences (TUMS). This centre is the main one for HSCT in Iran. The study was approved by the Ethics Committee of TUMS. 

**Patients**

This study included 173 patients (≥15 years old) who received either autologous or allogeneic SCT. Patients with the history of chronic gastrointestinal diseases such as peptic ulcer, Crohn's disease, ulcerative colitis were excluded from the study. During hospitalization, all patients were isolated in single bedrooms and had access to primary care services. Peripheral blood was used as the source of stem cells in all HSCT recipients. Patients’ characteristics (including age, sex, diagnosis, conditioning regimen, immunosuppressive medications as well as the type of HSCT) were recorded on a data collection form for each patient from the admission date until discharge. Each patient was evaluated three times per week. The day of HSCT was recorded as day 0 and all of the positive and negative numbers in this study indicate the days after and before HSCT, respectively.

**Conditioning regimens**

Various conditioning regimens were administered for patients before transplantation based on the underlying diseases and according to the hospital protocols. The summary of the regimens are mentioned in the result section. 

**Prophylactic measures**

In this centre, patients routinely received prophylaxis against nausea and vomiting. Additionally, anti‑infective prophylaxis against infections with candida, herpes zoster and pneumocystis carinii consisted of fluconazole tablet (100 mg BD), acyclovir tablet (200mg TDS) and sulfamethoxazole/trimethoprim (2 tablet 480mg BD) administered, respectively. Immunosuppressive agents for the prophylaxis against graft- versus- host disease (GVHD) consisted of cyclosporine plus methotrexate (MTX) or cyclosporine plus antithymocyte globulin (ATG) or cyclosporine plus MTX and ATG. 

**Prevention and management of mucositis**

All patients received two prophylaxis regimens in this centre from the date of admission. Prophylaxis regimen number 1 contained nystatin suspension 100000 U/ml (20 drops), chlorhexidine 0.2% (10 ml), 10 ml diluted povidone iodine (10 ml of povidone iodine in 1000 ml NS) and 10 ml diluted amphotericin B injection vial 50 mg (10 ml of amphotericin B in 1000 ml NS). Prophylaxis regimen number 2 contained nystatin suspension 100000 U/ml (20 drops) and diluted povidone iodine (10 ml). Both of the mouthwashes were ordered to be gurgled every 3 hours and were placed in patients’ rooms for daily usage.

Upon the diagnosis of mucositis, different treatment modalities were applied. In some patients either of the previously administered prophylactic regimens was continued. Some patients received a cocktail mouthwash consisted of diphenhydramine elixir 12.5 mg/5 ml (20 ml), aluminium magnesium hydroxide suspension (20 ml), lidocaine (10 ml lidocaine ampule 2% or 12 g lidocaine gel 2%) and 1 dexamethasone ampule of 8 mg. The remaining patients received a combination of the previous prophylactic regimens plus the cocktail mouthwash. To control the pain associated with mucositis, opiates were generally used. Tramadol, morphine and pethidine were also administered to reduce the pain.

**Assessment of mucositis**

We used the fifth-grade World Health Organization (WHO) oral toxicity scale^   20 ^ to evaluate the severity of mucositis (Table 1). Using the WHO scale in fact helps to incorporate the clinical symptoms determined by examination into the patients’ report regarding functions such as eating ability^   16 ^. The advantages of WHO scale is that it is valid, well known and can be easily used ^   8 ^. In this study, we categorized grade 1 and 2 of the scale as mild/moderate, while the grade 3 and 4 were defined as severe mucositis. 

For the assessment of mucositis, patients were visited regularly 3 times a week and at each visit the WHO scale was filled. The nurses were also asked regarding patients symptoms if necessary. 

**Table 1:** World Health Organization (WHO) scale for oral mucositis   ^(^^7^^)^

Grade 0	No oral mucositis
Grade 1	Erythema and soreness
Grade 2	Ulcers; able to eat solids
Grade 3	Ulcers; requires liquid diet (due to mucositis)
Grade 4	Ulcers; alimentation not possible (due to mucositis)

**Statistical analysis**

Continuous variables were expressed as mean ± standard deviation. Categorical variables were reported as percentages. Chi- square test was also applied for frequency analysis. The comparison of continuous variables was done using nonparametric Kruskal- Wallis test. Logistic regression was used to compare multivariate analysis, and the effect of important variables on the incidence of OM was assessed by adjusting other covariates.

## Results

**Patients’ characteristics**

One hundred and seventy-three patients consisting of 106 (61.3%) men were evaluated in this study, of whom 87(50.3%) and 86(49.7%) received allogeneic and autologous HSCT, respectively. Mean (SD) age of patients was 40.6±15.0 years. As expected, hematologic malignancies comprised the largest group of diseases leading to HSCT and among them leukaemia were the main diagnosis. 

**Conditioning regimens**

The most frequent chemotherapy regimens used for HSCT are as follows: in patients receiving allogeneic transplantation, busulfan + cyclophosphamide were used for the conditioning of patients suffering from Acute Myeloid Leukaemia (AML), Myelodysplastic Syndromes (MDS), Acute Lymphoblastic Leukaemia (ALL), Chronic Myeloid Leukaemia (CML) and Paroxysmal Nocturnal Hemoglobinuria (PNH). Melphalan was administered for Multiple myeloma (MM) and POEMS disease in autologous HSCT. The protocol of conditioning for Hodgkin and non-Hodgkin lymphoma in autologous HSCT consisted of carboplatin, cytarabine, etoposide and melphalan.

The conditioning regimen for patients with Aplastic Anaemia (AA), Thalassemia, Niemen Pick, Chronic Granulomatous Disease, Myelofibrosis, one patient with AML-M3 and another patient with AML was different from those mentioned in the Table 2. Patients with the exception of their type of HSCT were as follows: haploidentical HSCT for 5 patients with AML/ALL, allogeneic transplantation for 4 patients with MM and autologous HSCT for one patient with AML. 

**Prophylaxis against OM**

In our study, 70 patients (40.5%) received the first prophylaxis regimen, 89 patients (51.4%) received the second prophylaxis regimen and the remaining 14 patients (8.1%) did not have adherence to the use of the mouthwashes despite their availability. Baseline characteristics of patients who received the prophylactic mouthwashes are shown in Table 2.

**Table 2:** Characteristics of the study patients’ population

Patients characteristics	Value
**Sex No. (%)** Male Female	100(62.9) 59(37.1)
** Age groups No. (%)** (15-29 yrs.) (30-45 yrs.) (46-71 yrs.)	43(27) 50(31.4) 66(41.6)
**Diagnosis No. (%) ** Leukemia^1^ Multiple myeloma Lymphoma^2^ Others^3^	62(39) 58(36.5) 24(15.1) 15(9.4)
**Conditioning regimen No. (%) ** Bu(-6 to -3)+Cyclophosphamide(-2,-1) Melphalan VP16+CYT+Carboplatin+Melphalan Others^4^	56(35.2) 55(34.6) 24(15.1) 24(15.1)
**Immunosuppressant regimen in allogeneic HSCT No. (%) ** CY(-3 to discharge)+MTX(1,3,6,11) CY(-2 to discharge)+MTX(1,3,6) CY(-4 to discharge)+ATG(-3,-2,-1) CY(-2 to discharge)+MTX(1,3,6,11)+ATG(-3,-2,-1) Others^5^	52(66.7) 4(5.1) 4(5.1) 4(5.1) 14(18.0)
**Admission day ** Mean ± SD Min Max	-7.5±2.4 -21 -2
**Discharge day** Mean ± SD Min Max	16.5±4.5 11 39

Abbreviations: ATG: antithymocyte globulin, Bu: busulfan, CY: cyclosporine, CYT: cytarabine, MTX: methotrexate, VP16: etoposide, SD: standard deviation

1 Includes the number of patients diagnosed with Acute Myeloid Leukaemia (AML), Acute Lymphoblastic Leukaemia (ALL), and Chronic Myeloid Leukaemia (CML)

2 Includes the number of patients diagnosed with Hodgkin Disease and Non Hodgkin lymphoma

3 Other disease are as follows: Aplastic Anaemia (AA) (N=6), Thalassemia (N=3), Myelodysplastic Syndromes (MDS) (N=1), Niemen Pick (NP) (N=1), Chronic Granulomatous Disease (CGD) (N=1), Myelofibrosis (MF) (N=1), POEMS Syndrome (N=1) and Paroxysmal Nocturnal Hemoglobinuria (PNH) (N=1)

4 Busulfan+fludarabin, melphalan + fludarabin, cyclophosphamide alone, busulfan + cyclophosphamide + etoposide, busulfan + cyclophosphamide with different schedules other than the first regimen mentioned in the table

5 Other regimens are the same drugs with different timing schedule

**Mucositis**

Totally, OM was detected in 105 (60.7%) patients, of whom 96 (60.5%) had already been given prophylaxis regimens against OM. The mean (SD) age of the patients was 38.1±14.6 years. According to the WHO oral toxicity scale, 3 (1.9%), 47 (29.6%), 33(20.8%) and 13 (8.2%) of these patients experienced OM with the severity grade 1, 2, 3 and 4, respectively. The mean (SD) age of these patients was 38.6±14.9 years. Oral mucositis averagely lasted 9.9±3.9 days. The OM was started and ended on days 5.2±2.4 (mean±SD) and 14±3.6, respectively. 

The incidence of OM was significantly higher in female patients (42 (71.2%) versus 54 (54%), P=0.03). However, no significant difference was noted neither regarding the severity of OM (P=0.07) nor the duration (P=0.38) between the two genders.

**Figure 1:**
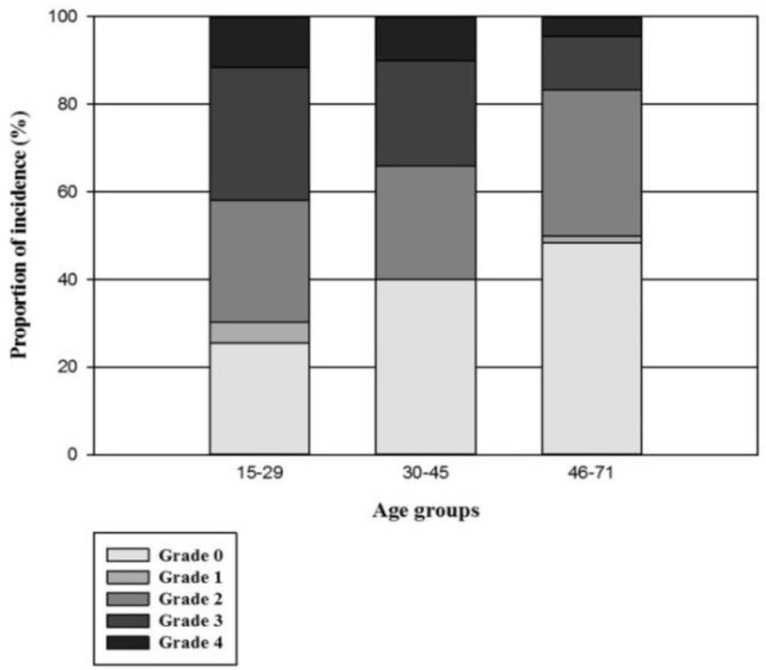
The proportion of incidence of OM and its severity in different age groups

We categorized the patients into three age groups and found that the incidence of OM had a decreasing trend with increasing age (32(74.4%), 30(60%) and 34(51.5%) in 15-29 years, 30-45 years and >45 years groups, respectively), but the trend was not statistically significant (P=0.06). The highest incidence of mild to moderate grades and the severe grade were observed in the eldest age group and the youngest, respectively (23 (34.8%) for mild/moderate grade and 18 (41.9%) with the severe grade, P=0.03). The average duration of OM was not different among the three age groups (P=0.08) (Figure 1). 

The most frequent conditioning regimens administered to 22 patients (64.7%) in the age group 15-29 and 28 patients (71.8%) in the age group 30-45 included busulfan and cyclophosphamide. In patients aged 46-71 years, melphalan was the most commonly used chemotherapy regimen in 51 patients (82.3%) (P<0.001). This shows that the incidence and severity of OM may depend more on the chemotherapy agents included in the conditioning regimens than on age.

The incidence and severity of OM in patients with different diagnoses are shown in Table 3. Among the diseases such as leukaemia, MM and lymphoma that were the leading causes of HSCT, leukaemia had the highest incidence and severity of OM among patients. 

We found that the mean duration of OM in patients with MM was significantly shorter than those with lymphoma and leukaemia (8.6±3.3 vs. 10.6±6.1 and 10.9±3.2 days, respectively P=0.007) (Table 3). When different conditioning regimens were compared, we noted that the incidence and frequency of severe grades of OM were significantly higher in patients who received the conditioning regimen containing busulfan plus cyclophosphamide (Table 3). Patients who received autologous HSCT experienced significantly lower incidence of OM, lower frequency of severe grades and also 1.4 days shorter duration of OM (Table 3). 

**Figure 2:**
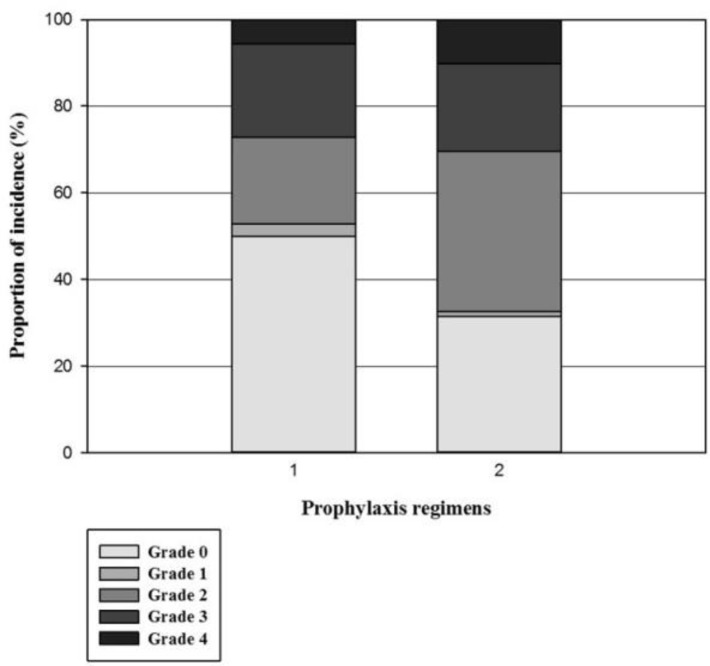
The proportion of incidence of OM with its severity among patients who received different prophylaxis regimens

We also found that the incidence of OM was significantly higher in patients who received the prophylaxis regimen No. 2 compared to the first regimen (68.5% vs. 50%, P=0.018). However, the severity and duration of OM was not significantly different between patients who received either of the prophylactic regimens (Figure 2). 

**Table 3:** Oral mucositis incidence, severity and duration based on patients’ characteristics

		**No of ** **Patients ** **(n= 159)**	**Incidence** **(n= 96)**	**P **_Value_	**Severity**		**P **_Value_	**Length**	**P **_Value_	
					**Mild/Moderate** **(n= 50)**	**Severe** **(n= 46)**				
**Diagnosis**										
Leukaemia		62(39%)	46(74.2%)	0.02	18(29%)	28(45.2%)	0.006	10.9±3.2		0.007
MM		58(36.5%)	27(46.6%)		17(29.3%)	10(17.3%)		8.6±3.3		
Lymphoma		24(15.1%)	13(54.2%)		10(41.7%)	3(12.5%)		10.6±6.1		
AA		6(3.9%)	2(33.3%)		1(16.7%)	1(16.7%)		4.5±0.7		
Thalassemia		3(1.9%)	3(100%)		1(33.3%)	2(66.7%)		11.3±3.5		
CGD		1(0.6%)	1(100%)		1(100%)	0		7.0		
MF		1(0.6%)	1(100%)		1(100%)	0		8.0		
NP		1(0.6%)	0		-	-		-		
POEMS		1(0.6%)	1(100%)		1(100%)	0		6.0		
PNH		1(0.6%)	1(100%)		0	1(100%)		6.0		
MDS		1(0.6%)	1(100%)		0	1(100%)		14.0		
Conditioning Regimens										
Bu(-6 to -3) + Cyclophosphamide (-2,-1)		56(35.2%)	42(75.0%)	0.02	16(28.6%)	26(46.4%)	0.003	10.8±3.3	0.008	
Melphalan (-2,-1)		55(34.6%)	28(50.9%)		18(32.7%)	10(18.2%)		8.5±3.5		
VP16 + CYT + Carboplatin (-2,-1) + Melphalan (-1)		24(15.1%)	13(54.2%)		10(41.7%)	3(12.5%)		10.6±6.1		
OM Prophylaxis Regimens										
1		70(44%)	35(50%)	0.02	16(45.7%)	19(54.3%)	0.344	9.69±4.276	0.43	
2		89(56%)	61(68.5%)		34(55.7%)	27(44.3%)		10.11±3.67		
Type of HSCT										
Autologous		81(50.9%)	42(51.9%)	0.02	29(35.8%)	13(16.1%)	0.001	9.17±4.356	0.01	
Allogeneic		78(49.1%)	54(69.2%)		21(26.9%)	33(42.3%)		10.57±3.39		
Types of Allogeneic HSCT	HLA Matched	67(42.1%)	45(67.2%)		19(28.4%)	26(38.8%)		10.4±3.6		
	Haploidentical	3(1.8%)	3(100%)		0	3(100%)		12.7±3.0		
	Unrelated	2(1.3%)	2(100%)		0	2(100%)		11.5±2.1		
	Other related	2(1.3%)	1(50%)		1(50%)	0		9.0		
	Other related & Haploidentical	2(1.3%)	1(50%)		0	1(50%)		10.0		
	Unrelated Mismatch	2(1.3%)	2(100%)		1(50%)	1(50%)		11.5±0.7		
Age(yrs.)										
15-29		43(27.1%)	32(74.4%)	0.06	14(32.6%)	18(41.9%)	0.032	10.31±4.659	0.08	
30-45		50(31.4%)	30(60%)		13(26%)	17(34%)		10.87±3.693		
46-71		66(41.5%)	34(51.5%)		23(34.8%)	11(16.7%)		8.82±2.979		

OM was observed in 9 (64.3%) of 14 patients who did not receive the prophylactic mouthwash. Four patients (50%) were women and 5 patients (83.3%) were men (P=0.12). Mild to moderate grades of OM were observed in 3 patients (21.4%) and the severe grade was observed in 6 patients (42.9%). The mean duration of OM was 11.11±2.76 days in this patient group.

**Management of oral mucositis**

**Figure 3:**
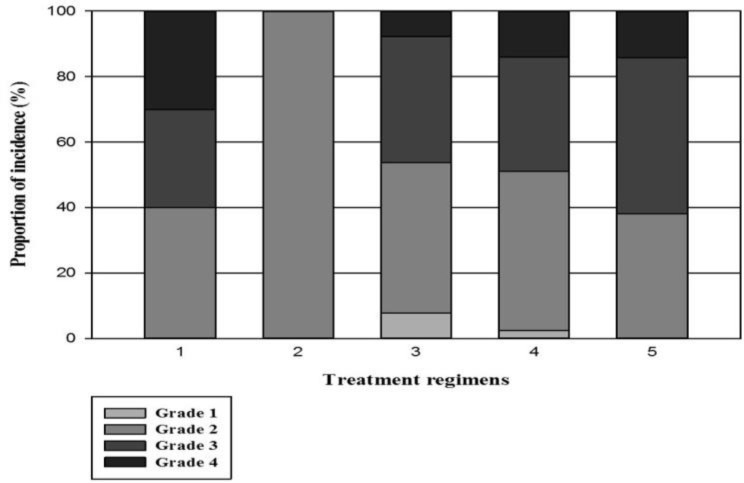
The proportion of incidence of OM and its severity in different treatment regimens

As mentioned previously, patients were categorized to five different scenarios regarding the treatment of mucositis (Figure 3). We found that the use of mouthwash cocktail with the application of prophylactic regimen No.2 was the most frequently administered therapeutic regimen which was used by 43(45.7%) of patients. Additionally, we found that the mean duration of OM and its severity was not statistically different among the five treatment regimens of OM (P=0.33) (Table 4). 

**Table 4:** Different therapeutic regimens used for the treatment of oral mucositis

**Treatment** **Regimens**	**No of** **patients** **with OM** **(n= 96)**	**Severity**		**P** **Value**	**Length** **of OM**	**P** **Value**
		**Mild/Moderate** **(n= 50)**	**Severe** **(n= 46)**			
1	10(10.6%)	4(40%)	6(60%)	0.31	9.2±2.4	0.32
2	7(7.4%)	7(100%)	0		8.0±2.9	
3	13(13.8%)	7(53.8%)	6(46.2%)		9.6±6.3	
4	43(45.7%)	22(51.1%)	21(48.9%)		10.6±3.9	
5	21(22.3%)	8(38.1%)	13(61.9%)		9.8±2.6	

1: cocktail mouthwash

2: prophylaxis regimen No. 2

3: prophylaxis regimen No. 1

4: cocktail mouthwash + prophylaxis regimen No. 2

5: cocktail mouthwash + prophylaxis regimen No. 1

Opioid analgesics were also administered to relieve pain of the OM. Tramadol, pethidine and morphine were used by 8(5%) patients (mean number of doses: 11 doses), 8(5%) patients (mean number of doses: 8 doses) and 3(1.9%) patients (mean number of doses: 10 doses) respectively. 

**Multivariate analysis**

In this study, we used a logistic regression analysis including variables such as age, gender, conditioning regimen, type of HSCT, the prophylactic mouthwash and diagnosis. We applied all of the factors without considering the significance of the variable in Chi- square test and found that by adjusting the variables, gender and the prophylactic mouthwash were significant variables affecting the incidence of OM. The results showed that female patients were more prone to develop OM than men (OR: 2.33, β: 0.848). Moreover, the prophylactic regimen 1 was significantly more effective in preventing the OM than the second regimen (OR: .47, β: -0.755) 

## Discussion

 In this study, we evaluated the current practice of a major HSCT centre in Iran regarding prophylaxis and management of OM and the determinant factors affecting OM in these patients. We found that several factors have a significant effect in the incidence of the OM but only few factors remain significant in multivariate analysis when adjusted for other variables (discussed latter). 

We noted that 60.7% of all patients developed some degree of OM. The incidence of this complication varies widely among studies. For example Wardley et al. reported that 99% of patients who received myeloablative conditioning regimens experienced OM^21^. In a multicentre study by Vagliano et al. in which 1841 HSCT patients were evaluated in Italy, 71.4% developed OM and the authors mentioned that the incidence of this complication was the lowest among other studies published before 2011^   8 ^. However, our results showed a lower incidence of OM among patients with different diseases. We noticed a lower incidence of sever grades of OM in patients with MM compared with Blijlevens et al. (17.3% vs. 46%). Additionally, the incidence of OM was lower in patients with lymphoma in our study compared with NHL in the aforementioned study (12.5% vs. 42%) ^   22 ^. As we mention later in this article, different factors might be responsible for this variation. 

We demonstrated that the incidence of OM as well as the duration and the frequency of experiencing severe grades of OM were significantly higher in patients who received allogeneic HSCT compared with those who received autologous HSCT. This significantly higher incidence  ^21^^,^^23^  and frequency of severe grades of OM following allogeneic HSCT ^   8 ^ have been pointed out by previous studies. 

When the first three frequent underlying diseases were compared, we noticed that the incidence as well as the frequency of developing severe grades of OM were significantly higher in patients with leukaemia. Additionally, we found that both the incidence and the frequency of severe grades of OM varied significantly among patients who received different conditioning regimens. In the current study, patients who received busulfan (-6 to -3) plus cyclophosphamide (-2,-1) experienced both the higher incidence as well as the more severe grades of OM. These findings were, to some extent, based on our finding regarding the underlying diseases. Since the mentioned conditioning regimen was the main regimen used for patients with leukaemia, it seems that the conditioning regimen has a determinant role. Similarly, Wardley et al. reported that the only significant variable that could affect the incidence of OM was the conditioning regimen^   21 ^. However, they reported that patients who received regimen including melphalan experienced the most severe grades of OM ^   21 ^. One of the reasons why the combination regimen of busulfan and cyclophosphamide were not the first regimen responsible for OM toxicity in their study might be attributed to the dose of the busulfan. In the Shariati hospital, the dose of busulfan was 4 mg/kg TDS, while in the Christie hospital it was 1mg/kg QID. Moreover, melphalan in their study was used for patients who received allogeneic HSCT ^   21 ^, while during our study period it was only used for autologous HSCT. 

We noted that the severity of OM differed significantly in patients with different age groups and those aged 46-71 years less frequently experienced the sever grades. However, neither the incidence nor the duration of OM in patients with different age groups showed a significant difference. The effect of age was evaluated in the study by Vagliano et al ^   8 ^. They categorized patients into paediatric, adults and elderly group and found that elderly patients experienced less severe grades of OM. However, they attributed the difference among the age groups more to the transplantation type and the level of treatment aggressiveness than age ^   8 ^. It should be noted that in our study the effect of age cannot be assessed separately from the conditioning regimen. The most frequently used conditioning chemotherapy agents for the youngest patients was busulphan + cyclophosphamide (64.7%) which was responsible for the highest incidence and more severe grades of OM, while the most frequent conditioning regimen used for the elderly patients was melphalan (82.3%) which was reported to be associated with the least incidence of OM compared with most widely used conditioning regimen in our study. We found that female patients were more prone to experience OM and the severe grades than men. The significant effect of gender on the incidence of OM in our study was also shown in the multivariate analysis. This finding was in keeping with previous studies such as the study by Vokurka et al ^   24 ^. Multivariate analysis of their randomized study in which povidone–iodine was compared with normal saline for the prevention of OM showed that female gender and conditioning regimen were the two independent factors predicting OM following autologous HSCT ^   24 ^. However, in another study on patients receiving allogeneic HSCT, the authors noted that based on the stepwise logistic regression, only the conditioning regimen was a significant factor affecting the incidence and severity of OM. They demonstrated that those patients who received busulfan + cyclophosphamide experienced significantly higher incidence of OM compared with ﬂudarabine + melphalan. However, despite the significant role of female gender as one of the predicting factors of OM in univariate analysis, multivariate analysis showed that only higher melphalan dose/kilogram of body weight was significantly associated with the incidence and severity of OM among the patients who received ﬂudarabine + melphalan ^   25 ^. In contrast, Salvador et al. in their study on patients receiving autologous HSC did not find that gender is a significant predictor of OM ^   26 ^. They reported that based on multiple logistic regression OM was significantly associated with the diagnosis of lymphoma, conditioning regimen (etoposide + melphalan), peak serum creatinine and using secondary prevention ^   26 ^. Similarly, Blijlevens et al. evaluated OM in patients with MM or NHL in several European centres in which patients received autologous HSCT conditioned by high-dose melphalan or carmustine, etoposide, cytarabine, and melphalan. They found that the only significant predictors of OM were the chemotherapy doses and diminished performance status ^   22 ^.

In the current study, we noted that two mouthwashes used for the prevention of OM contained nystatin, povidone iodine, chlorhexidine and amphotericin B (regimen 1) or nystatin and povidone iodine (regimen 2). It should be mentioned that using these agents are not in accordance with the high grade recommendations made by the 2014 MASCC/ISOO clinical practice guideline for the prevention of mucositis in cancer patients ^   27 ^. In the mentioned guideline, low-level laser therapy was recommended strongly for patients receiving high-dose chemotherapy for conditioning regimen before the HSCT ^   27 ^. However, due to the unavailability of this facility, none of the patients received this preventive measure in the current centre. Moreover, among the recommendations with weaker evidence, cryotherapy was suggested for those who received high-dose melphalan as a conditioning regimen ^   27 ^ which was not used in this centre (the highest dose of melphalan used for the conditioning of patients in this centre was 140 mg/m^2^)^. ^

Our results showed that not only the two prophylactic regimens differed significantly in terms of the incidence of OM based on the Kruskal Walllis test but also in the multivariate analysis this factor was a significant determinant of the incidence of OM. In this study, all of the patients received basic mouthwashes containing nystatin and povidone iodine. We found that the addition of chlorhexidine and amphotericin B to the prophylactic mouthwashes of one regimen made it significantly more effective in preventing OM. One important issue that needs to be taken into accounts is the compliance of patients. We noted that about 8% of patients in the current study were not adherent to using their mouthwashes. We excluded these patients in the analysis of comparing the two regimens due to the considerable effects that can be exerted to the results by ignoring this issue ^   28 ^. 

It should be noted that the comparison of different prophylactic measures are not reported in many observational studies  ^22^^, ^^23^^, ^^25^^, ^^29^ . However, the efficacy of different ingredients of the mouthwashes which were administered for OM prophylaxes in this centre has been evaluated in the literature. For example, Alvariño-Martín et al. in their review concluded that currently none of the interventions aimed to prevent or treat OM are considered to be the gold standard ^   30 ^. Moreover, Vokurka et al. in a randomized clinical trial compared normal saline with povidone-iodine diluted 1:100 in patients receiving autologous HSCT and found that neither of the overall incidence, severity and duration of OM were significantly different between the groups ^   31 ^. Additionally, despite a 30% reduction in the severity of mucositis achieved by using povidone-iodine compared with sterile water, Potting et al. did not recommend this agent and postponed the recommendation until stronger evince evolves ^   28 ^. In a systematic review published in 2006, Potting et al. also evaluated different mouthwashes used for the prophylaxis of OM caused by chemotherapy agents. They found that the studies do not support any significant positive effect in favour of chlorhexidine comparing with sterile water, or NaCl 0.9%. They concluded that using chlorhexidine mouthwash for the prophylaxis of OM is not justified and cannot be recommended ^   28 ^. In terms of using antifungal agents as the prophylactic mouthwashes in the systematic review by Potting et al., they reported that nystatin either alone or with chlorhexidine was not effective in preventing OM ^   28 ^. Additionally, in a clinical trial conducted by Epstein et al. oral suspension of amphotericin B was compared with nystatin in patients receiving HSCT. They showed that the severity of OM was not correlated with the colonization of Candida in oral cavity. However, the control of Candida colonization of oropharynx was better achieved by using theses oral suspensions ^32^. 

Based on the mentioned studies, it seems that adding chlorhexidine cannot be the main reason of why the two regimens were significantly different. Moreover, the combination therapy with nystatin and amphotericin B does not seem to be rational. However, the role of this combination to obtain the effective prevention of OM cannot be judged clearly. Since we did not have patients who receive amphotericin B as a sole agent and also we did not aim neither to evaluate the colonization nor the infection with Candida in this study, the effect of prevention of colonization or infection due to candida and the incidence of OM were not determined. Unfortunately, this issue was not pointed in the study by Epstein et al ^32^. To the best of our knowledge, previous clinical trials have not compared mouthwashes containing nystatin and amphotericin B with the same ingredients used in the current study. 

We recognized that in the current centre different management modalities were applied upon the diagnosis of OM. Chan et al. reported that the most frequent agents used in 40 institutions for the management of OM were diphenhydramine, lidocaine, magnesium hydroxide/aluminium hydroxide, nystatin and corticosteroids ^33^. They also highlighted the necessity of ingredients for the standardization^33^. The mouthwash cocktail in our study had similar ingredients. However, none of the 5 treatment approaches could significantly affect the duration of OM. Generally, in patients who develop OM, the condition is generally associated with considerable pain^27^. In our study, the most frequently prescribed medications for pain relief were tramadol and pethidine (in equal number of patients), followed by morphine on an as-needed basis. However, the recommended agent for this indication in the 2014 MASCC/ISOO clinical practice guideline was morphine through the patient-controlled analgesia^27^. 

**Limitations**

One of the limitations of our study was a wide range of patients with various underlying diseases, types of transplantations and conditioning regimens that made the comparison of some groups statistically difficult due to the small number of patients in the subgroups. Additionally, in this study, the number of patients who were not adherent to the preventive mouthwashes was not adequate to compare the three groups. Moreover, we compared different prophylactic mouthwashes in this study despite the fact that the current study was an observational study which has limitations compared to clinical trials in terms of assessment of the regimen efficacy. 

## CONCLUSION

 We noticed that the incidence of OM in patients undergoing HSCT is higher among patients with leukaemia, those who received conditioning regimen consisting of busulfan + cyclophosphamide, female patients, those who received the second prophylactic regimen and patients who underwent allogeneic HSCT. However, in multivariate analysis, only the female gender and the prophylactic regimen were significantly predictors of OM. Due to the significant effect of addition of amphotericin B and chlorhexidine to the mouthwashes to prevent OM, it could be considered the subject for future studies.
